# Improved classification accuracy of powdery mildew infection levels of wine grapes by spatial-spectral analysis of hyperspectral images

**DOI:** 10.1186/s13007-017-0198-y

**Published:** 2017-06-15

**Authors:** Uwe Knauer, Andrea Matros, Tijana Petrovic, Timothy Zanker, Eileen S. Scott, Udo Seiffert

**Affiliations:** 1 Biosystems Engineering, Fraunhofer IFF, Sandtorstr. 22, 39106 Magdeburg, Germany; 2 Leibniz-Institute of Plant Genetics and Crop Plant Research (IPK), OT Gatersleben, Corrensstraße 3, 06466 Seeland, Germany; 3 School of Agriculture, Food and Wine, The University of Adelaide, Waite Campus, PMB 1, Glen Osmond, Adelaide, SA 5064 Australia

**Keywords:** Grapevine, Powdery mildew, Hyperspectral, Image analysis, Infection

## Abstract

**Background:**

Hyperspectral imaging is an emerging means of assessing plant vitality, stress parameters, nutrition status, and diseases. Extraction of target values from the high-dimensional datasets either relies on pixel-wise processing of the full spectral information, appropriate selection of individual bands, or calculation of spectral indices. Limitations of such approaches are reduced classification accuracy, reduced robustness due to spatial variation of the spectral information across the surface of the objects measured as well as a loss of information intrinsic to band selection and use of spectral indices. In this paper we present an improved spatial-spectral segmentation approach for the analysis of hyperspectral imaging data and its application for the prediction of powdery mildew infection levels (disease severity) of intact Chardonnay grape bunches shortly before veraison.

**Results:**

Instead of calculating texture features (spatial features) for the huge number of spectral bands independently, dimensionality reduction by means of Linear Discriminant Analysis (LDA) was applied first to derive a few descriptive image bands. Subsequent classification was based on modified Random Forest classifiers and selective extraction of texture parameters from the integral image representation of the image bands generated. Dimensionality reduction, integral images, and the selective feature extraction led to improved classification accuracies of up to $0.998\pm 0.003$ for detached berries used as a reference sample (training dataset). Our approach was validated by predicting infection levels for a sample of 30 intact bunches. Classification accuracy improved with the number of decision trees of the Random Forest classifier. These results corresponded with qPCR results. An accuracy of 0.87 was achieved in classification of healthy, infected, and severely diseased bunches. However, discrimination between visually healthy and infected bunches proved to be challenging for a few samples, perhaps due to colonized berries or sparse mycelia hidden within the bunch or airborne conidia on the berries that were detected by qPCR.

**Conclusions:**

An advanced approach to hyperspectral image classification based on combined spatial and spectral image features, potentially applicable to many available hyperspectral sensor technologies, has been developed and validated to improve the detection of powdery mildew infection levels of Chardonnay grape bunches. The spatial-spectral approach improved especially the detection of light infection levels compared with pixel-wise spectral data analysis. This approach is expected to improve the speed and accuracy of disease detection once the thresholds for fungal biomass detected by hyperspectral imaging are established; it can also facilitate monitoring in plant phenotyping of grapevine and additional crops.

## Background

### Hyperspectral imaging

Hyperspectral imaging is a remote sensing technology that is becoming widely used in plant breeding, smart farming, material sorting, and quality control in food production [1], as well as identification of grapevine varieties from the air, detection and diagnosis of stresses caused by disease or nutrient imbalances and other applications in viticulture [2]. The generic behavior of the material to reflect, absorb, or transmit light is used to characterize its identity and even molecular composition. A hyperspectral camera records a narrowly sampled spectrum of reflected or transmitted light in a certain wavelength range and produces a high-dimensional pattern of highly correlated spectral bands per image pixel. Often, the direct relationship between this pattern and the target value, for example a nutritional or infection value, is unknown. In the simple case, exact spectral bands are known to correlate with the presence of certain chemical compounds. If such direct knowledge is unavailable, machine learning algorithms are used to learn a classification or regression task from labeled reference data [3].

Current sensor technology enables hyperspectral imaging at different scales. For imaging of small objects such as leaf lesions or seeds, frame-based hyperspectral cameras can be mounted on a microscope or line-scanning cameras can be equipped with macro lenses [4]. A common set-up for monitoring plants in the laboratory is a hyperspectral camera mounted to the side or above a conveyor belt or a translation stage [5]. While these set-ups have been partially adapted for outdoor measurements, for hyperspectral imaging of field trials, typically, vehicle-mounted hyperspectral cameras are used, for example on unmanned aerial vehicles (UAVs) [6]. The current limitations of this approach relate to the availability of lightweight sensors and loss of spectral and spatial resolution. Airborne and spaceborne hyperspectral imaging are options for the monitoring of production areas and large scale assessment of vegetation parameters.

### Approaches for analysis of hyperspectral data

Typically, the extraction of relevant information from hyperspectral datasets consists of the following steps. First, the hyperspectral data is normalized with respect to sensor parameters and illumination. Second, mapping between image pixels and known object positions is established, either by annotation of the acquired images or by automatically assigning coordinates (e.g. GPS measurements) to the image pixels. Third, preprocessing of images ensures extraction of meaningful entities by segmentation of objects (e.g. individual plants, leaves, fruits). As it is not possible to reliably detect individual objects in all cases, preprocessing can be restricted to suppression of the background information (e.g. soil surface). Low spatial-resolution of the hyperspectral dataset may require additional steps such as separation of the spectral information into components which characterize the mixture of different materials within the same pixel. In the remote sensing literature, this is known as spectral unmixing or endmember extraction [7].

Finally, the hyperspectral data (or derived measures such as indices) of a certain object or pixel is mapped to a target category/value provided by experts or laboratory analysis. Common indices such as Normalized Difference Vegetation Index (NDVI), Photochemical Reflection Index (PRI), Anthocyanin Reflectance Index (ARI) and others are sensitive [8, 9] but are not specific for plant diseases, which has necessitated the development of spectral disease indices (SDI) [10]. Disease indices are developed for specific host-pathogen combinations based on clearly defined reference data, but typically utilize a limited number of wavelengths and normalized wavelength differences. For example, in [10] a Powdery Mildew Index (PMI) for sugar beet has been proposed as $PMI=\frac{R_{520}-R_{584}}{R_{520}+R_{584}}+R_{724}$ where the $R_{xxx}$ denote normalized reflectances for certain wavelengths. Indices may fail due to changes in the properties of the biochemical background matrix.

Spectral Angle Mapping (SAM, [11]) takes all wavelength bands into account and is capable of discriminating between healthy tissue and tissue with powdery mildew disease symptoms at the microscopic scale. However, differentiation between sparse and dense mycelium remains difficult. As SAM does not weight the different wavelengths, the spectral angle is also sensitive to all changes in appearance even if they are unrelated to the symptoms (background matrix). In addition, large datasets for the dynamics of the pathogenesis of powdery mildew on barley have been investigated with data mining techniques [4]. Simplex volume maximization has been effectively used to automatically extract traces of the hyperspectral signatures that differ significantly for inoculated and healthy barley genotypes. While manual annotation of hyperspectral data by experts, as used in our study, provides accurate reference data, the approach of Kuska [4] effectively addresses the problem of large, automatically recorded hyperspectral datasets in time series analysis.

### Spatial-spectral segmentation with random forest classifiers

This paper addresses common challenges for the analysis of hyperspectral imaging data by investigating the classification performance of a novel approach to hyperspectral image segmentation. It is based on the tight coupling of Random Forest classifiers [12] with the integral image representation [13] of a dimensionality-reduced hyperspectral image.

There are two reasons for this approach. First, Random Forest classifiers are well established and combine fast and robust classification. Second, dimensionality reduction can bridge the gap between traditional pixel-wise classification of spectral information and texture-based image processing approaches for single band and color image segmentation which takes neighboring pixels into account and typically increases the accuracy of the image segmentation.

In general, image segmentation approaches can be roughly divided into pixel- [14] and region-based approaches [15]. Numerous approaches have been presented which treat image segmentation as a classification problem using different strong classifiers [16–19]. Other methodologies have been biologically motivated by principles of the human visual system [20, 21]. However, classification in high-dimensional feature spaces with the most sophisticated classification algorithms may not be an option for some approaches. For example, for many real-time image segmentation problems (online processing), either the number of features used must be limited to a few that are meaningful [22], a rather weak classification technique must be used, or both limitations are accepted in combination to meet the processing time constraints [23, 24]. Even if online processing of the data is not required, often the analysis results must be provided within a certain period to enable decision making in precision farming, disease control, nutrition management, and other applications.

Tree-based image segmentation has been reported [25], but for several years application seems to have been limited to certain fields, such as the segmentation of aerial or satellite imagery to identify land use. In recent years, Random Forest classifiers have been identified as a valuable tool in these fields as well as for related fields such as object detection [26]. New and demanding applications have led to several modifications and improvements of the original Random Forest approach to further improve the method and to match the application requirements. For example, Rotation Forest classifiers have been proposed as a method for improved classification of hyperspectral data [27] by adding transformations of the input feature space and hence contributing to the diversity of ensemble decisions. Also, semi-supervised sampling has been reported to improve the segmentation performance of conventional Random Forest classifiers [28].

### Feature relevance

Identification of relevant features for classification is a crucial task for effective processing as well as for a better understanding of the problems and their solutions. In [29] the performance of different feature selection approaches and classifiers for tree species classification from hyperspectral data obtained at different locations and with different sensors was reported. The authors conclude that the selection of 15–20 bands provides the best classification results and that the location of the selected bands strongly depends on the classification method. However, best classification results for all datasets have been obtained with Minimum Noise Fraction (MNF) transformation and selection of the first 10–20 principal components of MNF as input features for classification. In [30] the input feature space is extended by parallel extraction of spectral and spatial features. Then, a so-called hybrid feature vector is created and used for training of a Random Forest classifier. Finally, results are improved by imposing a label constraint which is based on majority voting. Other recent developments in hyperspectral image classification are reviewed in [31]. The authors present a Statistical Learning Theory (SLT) based framework for analysis of hyperspectral data. They highlight the ability of SLT to identify relevant feature subspaces to enable the application of more efficient algorithms. The review categorizes existing spatial-spectral classification approaches into spatial filters extraction, spatial-spectral segmentation, and advanced spatial-spectral classification.

### Scope of the spatial-spectral segmentation approach

In this paper we present an improved texture-based spatial-spectral approach to hyperspectral image classification which can potentially be applied to images from all available scales. This approach addresses the problem that pixel-wise processing of spectral data, even of derived information such as SDI, does not incorporate information about the spatial variation of the spectral properties of healthy and diseased material. Hence, taking this variation into account aims to improve classification accuracies for prediction of disease severity.

As a model system we selected the classification of powdery mildew infection levels of Chardonnay grape bunches, because the current approach of visual assessment of infection levels (% of surface area affected of a bunch) is subjective. Many Australian wineries use a rejection threshold of 3–5% surface area affected by powdery mildew based on visual assessment [32]. Thus, objective assessment of disease-affected bunches and quantification of pathogen (*Erysiphe necator*) biomass are required. Hyperspectral imaging was investigated as a means of detecting powdery mildew-affected bunches at the beginning of bunch closure, after routine assessment of disease in the field. Powdery mildew is more readily assessed by visual inspection at this stage than later in bunch development, providing a proof of concept for subsequent investigation of the disease on bunches closer to harvest.

We acquired hyperspectral images from powdery mildew affected and non-affected Chardonnay grape bunches. After preprocessing, the data sets were reduced in dimensionality by means of Linear Discriminant Analysis (LDA) to retain only a few highly descriptive image bands. Subsequent application of Random Forest classifiers and selective extraction of texture parameters led to improved classification accuracies for powdery mildew infection levels and, hence, disease severity level prediction (SLP) of wine grapes.

## Methods

### Plant material and fungal biomass

Grapes from a non-commercial vineyard (Waite Campus, University of Adelaide, South Australia) (E 138° 38′ 3.844″, S 34° 58′ 3.111″) were used in this study. In-field assessment of powdery mildew on vines was conducted according to [33]. Subsequently, 10 visually healthy and 20 bunches naturally infected by *Erysiphe necator* with no signs of other diseases and/or abiotic/biotic damage, were selected from Chardonnay vines (*Vitis vinifera L.*, clone I10V1). Bunches were collected at the lag phase of berry development (i.e. when berry growth is halted and the seed embryos grow rapidly), otherwise described as growth stage E-L 30-33 (beginning of bunch closure) [34] when total soluble solids had reached 5° Brix (December 4, 2014).

Bunches were assessed in laboratory conditions using a magnifying lamp and assigned to three categories: visually healthy, infected, and severely diseased. Bunches designated severely diseased were considered likely to have been infected at E-L 23-26 when grape clusters are highly susceptible to the pathogen. Berries on those bunches were significantly lighter ($0.53 \pm 0.045$ g, $p = 0.03$) and slightly smaller ($9.92 \pm 0.34$ mm) than berries on healthy bunches (weight $0.75 \pm 0.045$ g; diameter $11.20 \pm 0.34$ mm). However, morphology of all bunches was similar, regardless of powdery mildew status. After hyperspectral imaging of the upper and lower surface of each bunch, bunches were stored at $-20\,^{\circ}\hbox {C}$. Each surface of the frozen bunch was matched with the corresponding annotated reference image (Fig. 10) and berries were detached and grouped according to bunch and surface (30 bunches $\times$ 2 surfaces). The 60 samples were homogenized separately, then DNA was extracted using a Macherey-Nagel NucleoSpin® Plant II Kit and quantified using a QuantiFluor® dsDNA System. A modified duplex quantitative polymerase chain reaction(qPCR) assay using a TaqMan® MGB probe ($\hbox {FAM}^{\mathrm{TM}}$ dye-labelled) was used to quantify *E. necator* biomass [35]. Reaction efficiency was assessed by generating a standard curve for *E. necator* and absolute quantification of *E. necator* biomass was achieved using the standard curve. The number of copies of the amplified *E. necator* DNA fragment per conidium was calculated based on the DNA extracted from a known number of *E. necator* conidia. Consequently, the number of copies of the *E. necator* DNA fragment obtained for the DNA extracted from 100 mg of berry tissue was expressed as number of *E. necator* conidia and then corrected for the average weight of berries for each bunch. Log-transformed data is presented (Fig. 4).

### Hyperspectral imaging

**Fig. 1:**
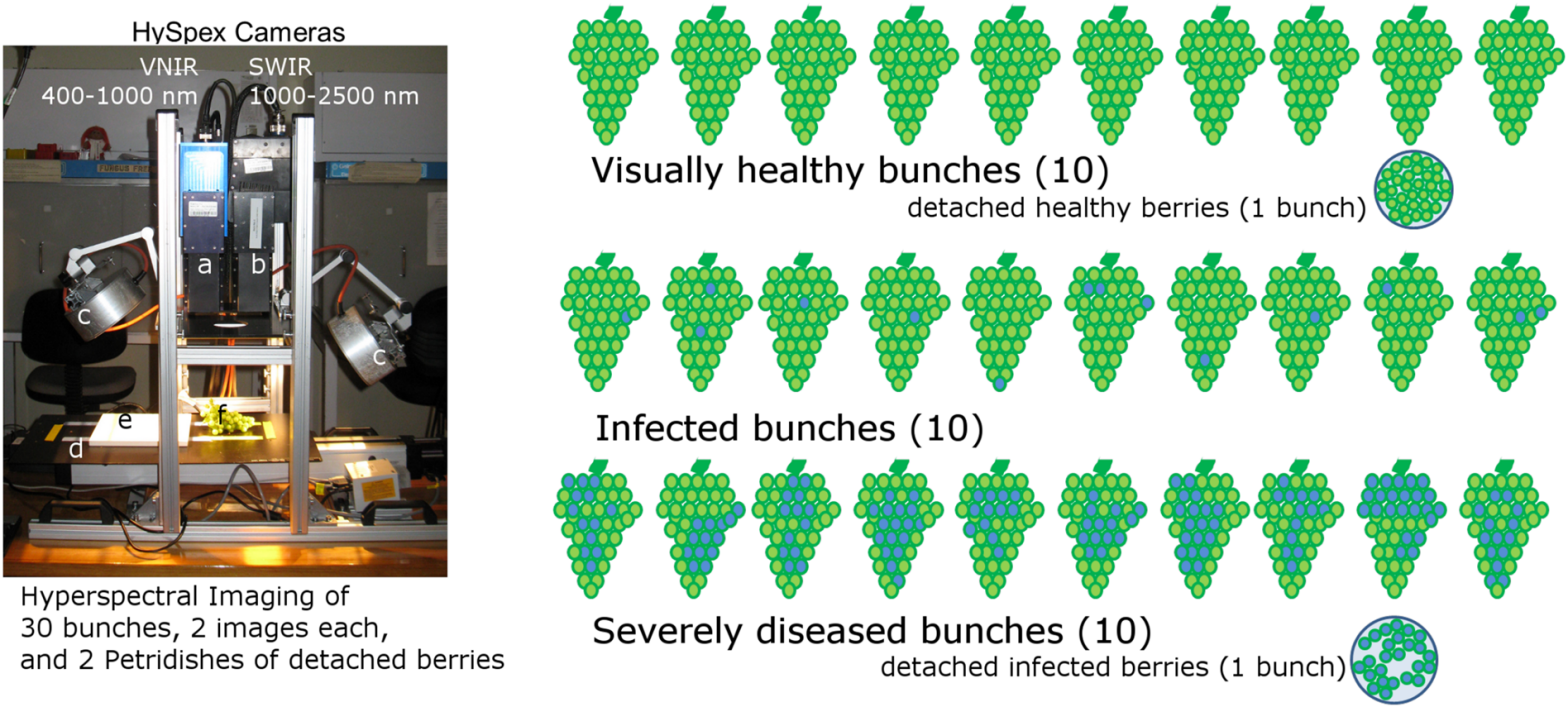
Overview of the measurement set-up and the experimental design. The measurement set-up consists of two hyperspectral line scanning cameras for VNIR (**a**) and SWIR (**b**) wavelength range, artificial broadband illumination (**c**), and translation stage with stepper motor (**d**). Hyperspectral images of PTFE reference plate (**e**) and 30 bunches (**f**), visually assigned to three categories (visually healthy, infected and severely diseased, *blue shading* represents powdery mildew), were recorded in laboratory conditions. Berries of two bunches were detached to be used as reference data for classifier training

Figure 1 provides an overview of the measurement set-up and the experimental design. For the hyperspectral image acquisition, samples of grapes were positioned along with a standard optical PTFE (polytetrafluoroethylene) calibration pad on a translation table. Spectra were acquired either from the visible and near-infrared range (VNIR) of 400–1000 nm at 3.7 nm resolution or from the short-wave infra-red range (SWIR) of 970–2500 nm at 6 nm resolution yielding a 160 dimensional or 256 dimensional spectral vector per pixel, respectively. Hyperspectral images were recorded using HySpex VNIR 1600 (VNIR camera) and HySpex SWIR-320m-e (SWIR camera) line cameras (Norsk Elektro Optikk A/S). The VNIR camera line has 1600 spatial pixels. Spectral data along this line can be recorded with a maximum frame rate of 135 frames per second (fps). The SWIR camera line has 320 spatial pixels. Spectral data can be recorded with a maximum frame rate of 100 fps. Radiometric calibration was performed using the vendor’s software package and the PTFE reflectance measure.

As part of the controlled environment, artificial broadband illumination was used as the only light source. Before the recordings started, two custom made lamps were adjusted to focus the light to a line overlapping the fields of view (FOV) of the hyperspectral cameras.

**Fig. 2:**
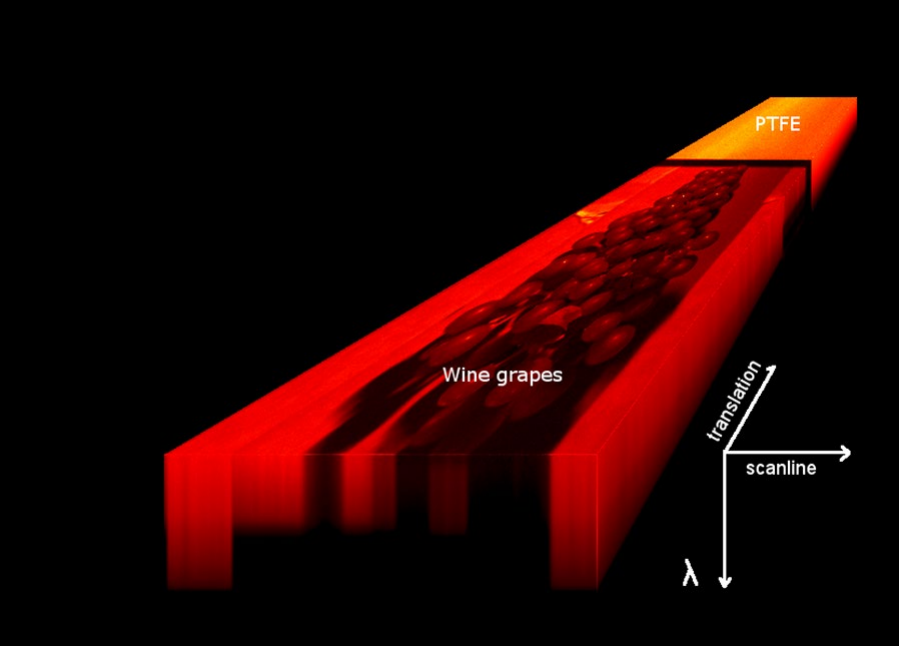
Hyperspectral image. Visualization of a hyperspectral image cube with grape bunch, PTFE reference plate, and background materials. The hyperspectral image consists of different layers which directly correspond to the reflection of narrow wavelength bands. The PTFE reference plate is calibrated and used for data normalization

Two hyperspectral images containing either only visually healthy or only severely diseased detached berries, manually dissected from two bunches, were recorded. Those images alone were used for SLP model development. Next, 60 images of two sides of 30 complete bunches were recorded, comprising 10 visually healthy bunches, 10 powdery mildew infected bunches, and 10 severely diseased bunches. These images were used to assess the accuracy of the SLP method under realistic conditions. Results of qPCR analysis of berries detached from all bunches served as reference values. Figure 2 illustrates the scanning result. It shows the hyperspectral data cube with two spatial and the spectral dimension. Each horizontal slice corresponds to a single wavelength image. The 1000 nm band of the VNIR camera is plotted on top.

### Development of disease severity level prediction models

**Fig. 3:**
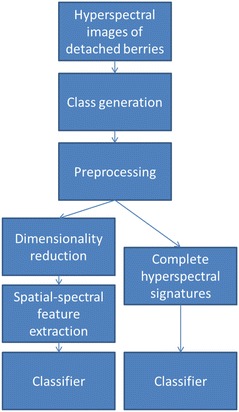
Systematic approach and development of powdery mildew detection models. Based on hyperspectral images of visually healthy and severely diseased detached berries a dataset containing spectra of both classes is generated. Two different feature spaces are investigated for classification of spectral data; first, dimensionality reduction with subsequent spatial-spectral feature extraction and second, classification of complete spectral signatures. The path on the right corresponds to the first row of Table 1, whereas the *left hand side* corresponds to the remaining rows

Figure 3 summarizes the approach for the development of models for SLP based on pixel-wise powdery mildew detection. For the development of prediction models and initial tests of parameters, only the small subset of images obtained from detached berries was used. First, this facilitates the generation of class information as the image contains either severely diseased or healthy berries. Second, the derived models can later be tested with the complete set of hyperspectral images. This ensures independent samples for validation of the approach. Preprocessing of the spectral data was undertaken to compensate for the specific contributions of the sensor as well as the illumination to the measured signal.

#### Image preprocessing

The preprocessing of hyperspectral images consists of the following steps:

1. Conversion from raw images (photon count, digital numbers) to radiance (at sensor)
2. Conversion from radiance (at sensor) to reflectance (at surface)
3. $L_2$-normalization (spectra are treated as vectors and normalized to have equal length)
4. Dimensionality reduction

Dimensionality reduction aims to achieve the following goals:

1. Reduction of computational costs
2. Avoid problems inherent in dimensionality (known as the curse of dimensionality [36] and particularly Hughes phenomenon [37] in machine learning and computational intelligence)

We implemented different options for dimensionality reduction:

1. Canonical band selection (inspired by human perception and bands of other existing imaging sensors),
2. Relevance-based band selection based on importance histograms,
3. Synthesis of orthogonal bands based on Principal Component Analysis (PCA),
4. Target class specific synthesis based on adapted data sampling before PCA,
5. Synthesis of orthogonal bands based on LDA.

Depending on the classification task at hand, each option provides a different trade-off between transformation speed and discriminative power of the original spectral data.

For canonical band selection the image bands used by the software PARGE (ReSe Software) were selected. For VNIR cameras such as NEO HySpex VNIR 1600, the red-channel of the resulting RGB-image was mapped to the 651 nm band, the green-channel to 549 nm, and the blue-channel to 440 nm. Another option for canonical band selection is close infrared (CIR), where the three channels were mapped to the 811, 640, and 498 nm bands, respectively. In the short-wave infrared, the following mapping was used: (1081, 1652, 2253 nm).

The relevance-based band selection was based on supervised pixel-wise classification of spectral information with Random Forest classifiers. During the construction of a decision tree, many different optimizations (with respect to a measure of information gain) take place for feature selection. Hence for each classification, the tree nodes visited were checked for which feature (band) was used to create a histogram of band importance. Finally, the three highest ranked bands were selected.

PCA was used to derive a new orthogonal base of the original feature space. The resulting bands represent linear combinations of all original bands. Random subsets of spectra were used to calculate the projection matrices. For target class-specific PCA the input spectra were sampled from predefined pixels only. Closely related is the application of LDA for deriving a task-specific projection.

#### Spatial-spectral classification

Our approach for texture-based classification (spatial component) relies on the data structure of integral images [13]. This representation enables a cache-like fast look-up of feature values for arbitrary rectangular image regions of a single image band. Three base features are used, which require calculation of three integral images per image band:

1. Mean intensity
2. Standard deviation
3. Homogeneity

The choice of the base features is motivated by their known support for the integral image representation [13, 38].

These base features are calculated for 25 differently sized squared image blocks centered on the current pixel and all image channels (of the dimensionality reduced hyperspectral image) separately. Here, a 225-dimensional ($3\times 3\times 25$) feature vector is used per pixel. Even if the dimension of the feature vector is approximately the same as for the spectral data, each feature now consists of a spatial (mean, standard deviation or homogeneity of rectangular image area) and a spectral component (from PCA, LDA or band selection).

In the training phase, feature vectors were selected at random locations within the image. Class labels were assigned based on given reference data. Next, a modified Random Forest classifier was trained. In contrast to the default Random Forest classifier, each tree node holds additional information which is needed to quickly access the tested feature from the set of integral images. Hence, there is no need to calculate a full feature vector in the application phase of the model. For each pixel only a subset of dimensions of the feature space must be calculated. This speeds up the classification process. A significant reduction in the time needed for calculation of features can be obtained for single decision trees (in the order of $\log _2{N}$, where *N* is the total number of considered features) and Random Forests with a small number of trees. A related investigation of the trade-off between classification accuracies, ensemble size, and number of features used for different hyperspectral classification tasks can be found in [39].

### Cross-validation procedure

N-fold cross-validation was used to calculate an estimate for the classification accuracy (N = 10 was used for all experiments). The training data was randomly partitioned into 10 groups (folds) of equal size. This means that each feature vector was assigned to only one of the folds. While $N-1$ folds were used to train a classification model, the remaining fold was used to test the accuracy of the resulting model. This was repeated N times. The average accuracy and the standard deviation of the N classification models were then compared.

## Results

### Fungal biomass

**Fig. 4:**
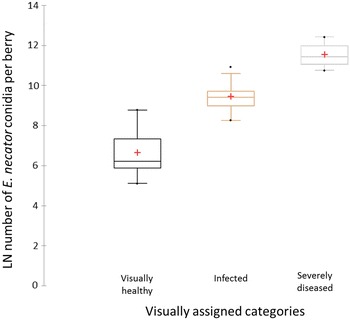
Quantitation of *Erysiphe* *necator* biomass in Chardonnay grape bunches. *Boxplot* of *E. necator* biomass as measured by an *E. necator*-specific qPCR assay of bunches assigned to three visual categories (visually healthy, infected, and severely diseased). Four bunches or 40% of scanned bunch profiles of visually healthy bunches were confirmed to be pathogen-free according to qPCR

The differentiation between visually healthy, infected and severely diseased bunches proved to be accurate for the majority of bunches (75%) based on fungal biomass (via qPCR) as reference (Fig. 4). Of the visually healthy bunches, four were negative in the qPCR assay so the fungus was not detected on either side of the bunch. However, the fungal biomass among the remaining six visually healthy bunches varied considerably. Fungal biomass from infected and severely diseased bunches showed less variation. Maximum fungal biomass for visually healthy and infected bunches overlapped with biomass for infected and severely diseased bunches, respectively (Fig. 4). Overlap in fungal biomass was more evident for visually healthy and infected bunches than for infected and severely diseased bunches. This indicates that bunches visually assessed to be healthy had colonized berries hidden within the bunch, sparse mycelial growth missed under the magnifying lamp or that airborne conidia had landed on the berry surface. Uneven distribution and density of *E. necator* mycelium and conidiophores on berries in infected bunches is likely to have caused the overlap in fungal biomass between infected and severely diseased bunches (Fig. 4).

### Dataset

**Fig. 5:**
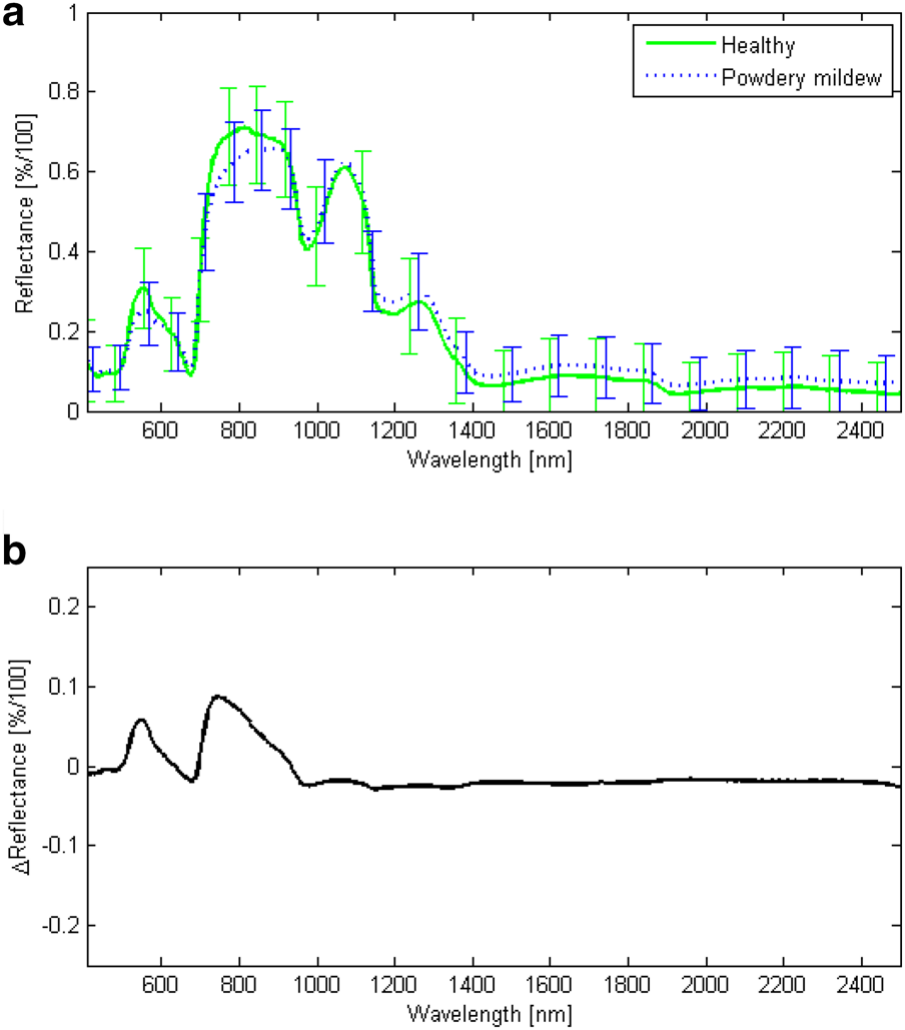
Illustration of reflectance spectra. Spectral signatures of healthy detached berries and detached berries with severe powdery mildew infection (**a**) and the differences between mean spectra of healthy and diseased berries (**b**). The standard deviations of the spectral signatures are shown as *error bars* in **a**. Spectrally localized differences are observed in the *green peak region* (550 nm) of the spectra and just above the *red edge region* (680–730 nm). Throughout the shortwave infrared region a shift between the mean spectral signatures occurs due to higher reflectance of the diseased berries

The dataset consists of 60 hyperspectral images corresponding to two scans (top and bottom view) of 30 bunches (see Fig. 1). From two of these bunches, 128 visually healthy and 136 severely diseased berries were selected and detached for recording of an additional dataset for classifier training and initial validation. Detached berries were arranged in Petri dishes and two additional hyperspectral images were recorded which contained either severely diseased or healthy berries. Furthermore, the small time gap between the two recordings ensured constant conditions for the measurements. Figure 5 shows the mean spectra as well as the standard deviations obtained from these reference images for healthy and severely diseased detached berries. Here, the spectral signatures of each image pixel have been normalized with respect to the reflectance of the PTFE calibration pad.

**Fig. 6:**
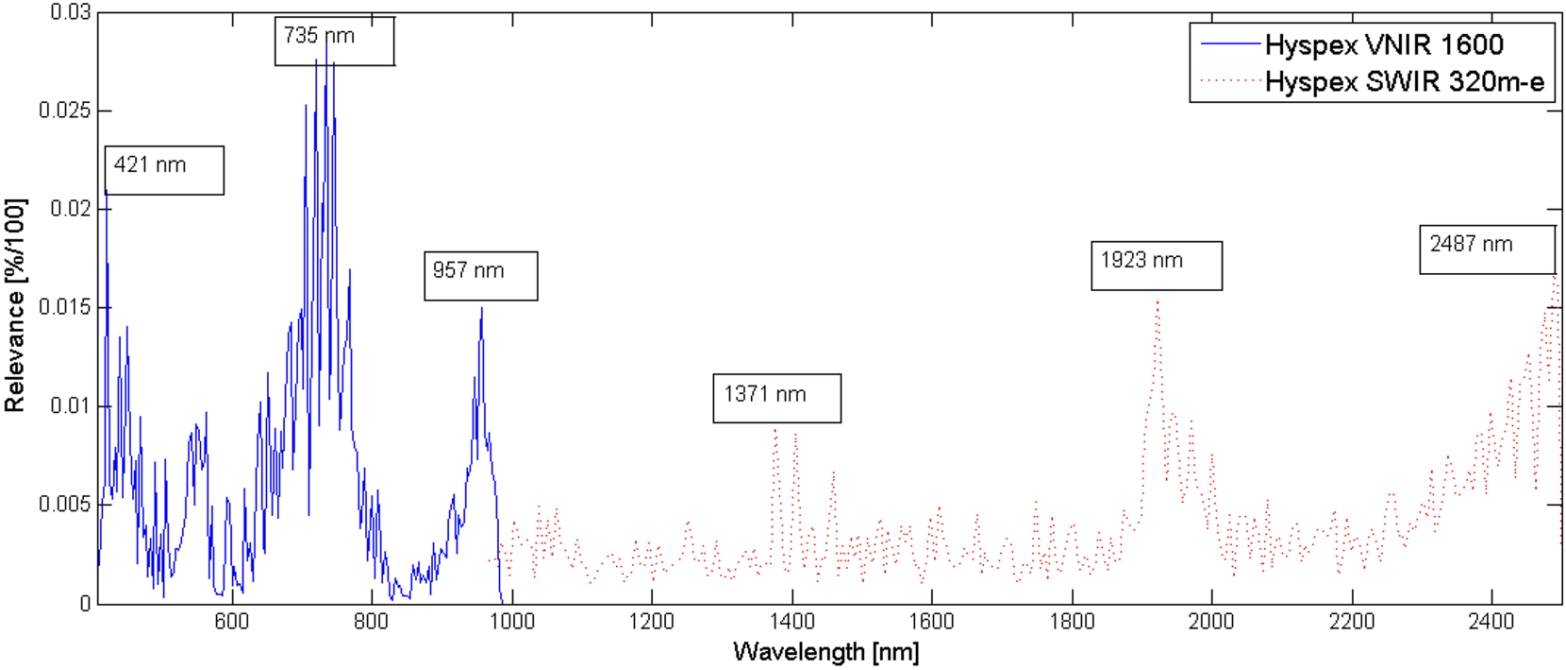
Relevance spectrum. Relevance of the individual spectral bands was derived from the structure of the Random Forest classifiers. More relevant wavelength features are used more often and hence contribute more to the final decision. The images of the two hyperspectral cameras have been processed independently and result in the blue and the red relevance profile, respectively. For each camera a number of highly relevant bands are found. Three local maxima in the relevance profiles are highlighted. Limiting classification to only the three highlighted relevant wavelengths yields mean accuracies of 0.98 (VNIR camera) and 0.99 (SWIR camera) for detached berries and in combination with textural features extracted from these image bands

For validation of the proposed spatial-spectral approach these spectral signatures have been used to train a reference Random Forest classifier. Figure 6 shows the relevance profile derived for individual wavelengths within the classification process. For the dimensionality reduction step in spatial-spectral segmentation, one option is to select the most relevant bands from this result. Additionally, a number of low-dimensional representations of the hyperspectral images have been derived to investigate the classification performance of the spatial-spectral image segmentation approach in different feature spaces.

### Classification models

In order to maintain speed of the proposed segmentation algorithm, dimensionality reduction is the first processing step. The fastest and simplest approach is focusing on a few (typically three) predefined image bands and skipping processing of all the others. Several such selections, for both VNIR and SWIR wavelength ranges, are compared to the more sophisticated reduction methods in Table 1. The mean accuracy values and their standard deviations are given for 10-fold cross-validation experiments for random sets of 4000 pixels from two training images (containing healthy and infected grapes). Results indicate that successful classification is possible in both wavelength ranges. However, with an accuracy of 0.98, pixel-wise spectral classification in VNIR performs significantly better than in SWIR (accuracy 0.85). The introduction of texture features by the spatial-spectral classification approach can nearly compensate for the effects of dimensionality reduction for all variants and improve classification accuracy to 0.99 (especially in the SWIR region this is a significant improvement). The transformations investigated for reduction of dimensionality (PCA, LDA, adaptive PCA) incorporate all image bands, potentially minimizing the loss of information inherent in dimensionality reduction, while band selections (Custom, RGB, CIR, SWIR) have been tested to exploit the potential of less expensive standard (RGB, SWIR, CIR) or customized (Custom) camera systems. The customized band selection was based on the analysis of the relevance of individual bands for a Random Forest classifier. To obtain a measure of relevance, during classification all nodes visited in the decision trees within the Random Forest voted for the corresponding feature. Three local maxima of the relevance curve were then selected. A threshold ensures a minimum distance of 20 bands between selected local maxima.

**Table 1:** Classification accuracy using different dimensionality reduction methods

Feature space	Bands	VNIR	SWIR
Normalized spectral*	All	0.980 ± 0.006	0.853 ± 0.027
PCA	All	0.968 ± 0.008	0.999 ± 0.002
Adaptive PCA	All	0.969 ± 0.007	0.996 ± 0.004
Custom	3	0.981 ± 0.008	0.997 ± 0.003
RGB	3	0.972 ± 0.009	–
CIR	3	0.971 ± 0.009	–
SWIR	3	–	0.999 ± 0.003
LDA	All	0.998 ± 0.003	0.998 ± 0.005

Principal Component Analysis (PCA) and standard band selections (RGB, CIR, SWIR) are compared to adaptive reduction methods. Adaptive PCA is based on stratified sampling based on class labels, custom band selection is based on relevance profiles and uses only three most relevant individual bands, while Linear Discriminant Analysis (LDA) is used to find an optimal subspace projection of the data

* Pixel-based segmentation of normalized spectra as reference, all other are spatial-spectral-based

Table 2 shows the investigation of block size (of spatial-spectral features) *vs* classification accuracy. LDA-based reduction and two predefined band selections (denoted as RGB and SWIR) have been compared. The results, especially for SWIR, indicate that good performance is already achieved with small maximum block sizes. The baseline accuracy for individual pixel classification (block size 1 pixel) is 0.78 for the VNIR camera and 0.94 for SWIR camera. This result shows the value of using disease-specific LDA based projection to constitute a low-dimensional representation for further processing. Classification accuracies for a representation by three default bands from the VNIR camera (RGB) or SWIR camera are 0.76 and 0.62 (block size 1 pixel), respectively. By increasing the maximum block size, additional features (mean, standard deviation, and homogeneity of intensity distribution) are taken into account which are not defined for a single pixel. For a maximum block size of 100 × 100 pixels in the VNIR camera image, which corresponds to the approximate size of a single berry, an accuracy of 0.99 is achieved. For image blocks of 20 × 20 pixels of the SWIR camera, accuracy of 0.99 was achieved also. As the sample in this experiment consists of detached berries which are covered by mycelium, the block size and classification performance can be further increased. However, in practice early detection of a powdery mildew-affected surface requires the use of small block sizes (to detect small infection spots).

**Table 2:** Classification accuracy versus maximum block size for spatial feature extraction

	1	5	20	50	100
RGB	0.767 ± 0.013	0.938 ± 0.014	0.952 ± 0.011	0.964 ± 0.01	0.972 ± 0.009
LDA VNIR	0.782 ± 0.016	0.865 ± 0.016	0.951 ± 0.015	0.984 ± 0.007	0.998 ± 0.003
SWIR	0.617 ± 0.027	0.729 ± 0.047	0.872 ± 0.019		
LDA SWIR	0.948 ± 0.017	0.986 ± 0.009	0.993 ± 0.006		

With increasing maximum block size (from left to right) a gain in accuracy was achieved by introducing additional spatial-spectral features. Due to the different resolution of the cameras for the VNIR and SWIR domains, 100 × 100 pixels in the VNIR camera image match 20 × 20 pixels in the SWIR camera image of the same bunch. These two block sizes correspond to the approximate size of a single berry in the measurement set-up used. The rows RGB and SWIR refer to spatial features derived from selected bands, while rows LDA VNIR and LDA SWIR refer to texture features derived from projected images. For the VNIR wavelength range the spatial component contributes most to the accuracy gain, while in the SWIR wavelength range classification of spatial features from projected images outperformed classification based on spatial features from selected bands. Even by introducing only a few spatial features (maximum block size 5 pixels), a significant gain in classification accuracy was observed. Due to the different spatial resolution of VNIR and SWIR images, which is related to the different number of pixels and pixel sizes, the increase of the block size was limited to the approximate size of a single Chardonnay berry (VNIR 100 × 100, SWIR 20 × 20 pixels)

Classification results correspond to the mean spectra plotted in Fig. 5 and with results from the literature [10]. Especially, in the SWIR domain the mycelium leads to a shift of the spectral signatures due to a higher reflectance over the complete wavelength range between 1000 and 2500 nm. While such a shift has been reported for powdery mildew-affected sugar beet in VNIR, the mean spectra show a different performance for grapes. We observed a reduced reflectance at the green peak region (550 nm) as well as in the plateau region after the red edge (750–900 nm). This is due to the high reflectance of healthy grapes compared to the reflectance of healthy leaves, which has been the subject investigated in previous studies [10, 11].

### Severity level prediction

Having an automated inspection system either in quality control or in plant phenotyping in mind, it is not feasible to scan detached berries and the scanning of complete bunches is much more challenging. An automated inspection system would deliver a score corresponding to the severity level or surface area affected by powdery mildew. Despite the promising results of cross-validation experiments within the training datasets (detached berries), the spatial-spectral classification of the complete bunch images yields different results. Their 3D structure imposes some additional problems with shadow and the focal plane compared to the recording of selected individual berries which were used for model generation. So far, in the SWIR wavelength range successful classification was not possible using the independently generated models for detached berries. Obviously, the observed shift in the hyperspectral signatures (Fig. 5) is the dominating discriminating feature and is impossible to detect in the presence of the aforementioned factors.

**Fig. 7:**
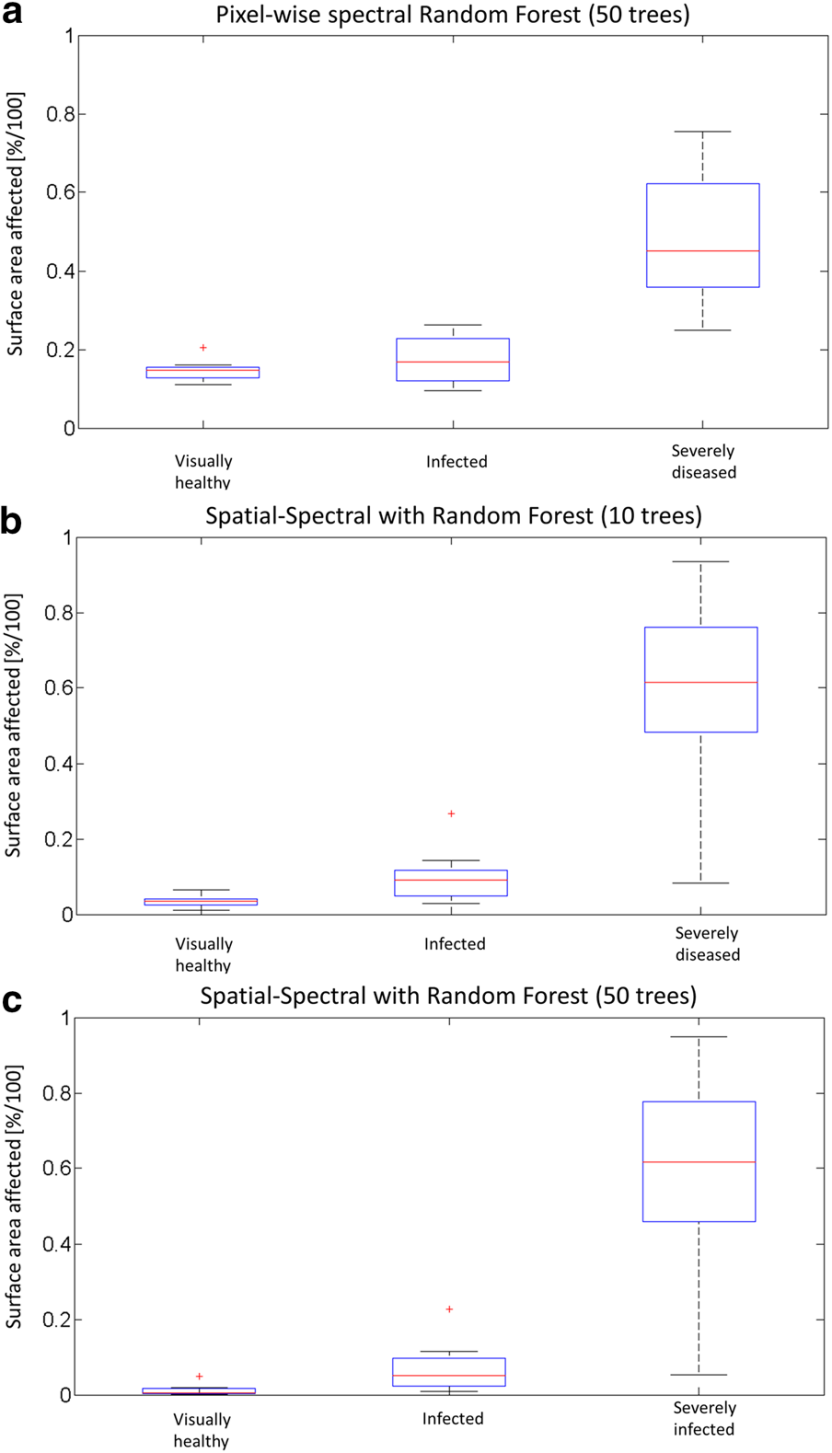
Classification accuracy of intact bunches depending on random forest classifier complexity. *Boxplot* of the predicted surface area affected for the three main categories of the experiment based on pixel-wise segmentation of LDA projected hyperspectral images (VNIR only). **a** Pixel-wise pure spectral classification with Random Forest, **b** texture-based spatial-spectral segmentation with 10 trees versus **c** Random Forest with 50 trees. Severely diseased bunches can be detected with high accuracy, while discrimination between healthy and infected is challenging in a few cases. Classification accuracy increases with the complexity (number of decision trees) of the Random Forest classifier. Results of the analysis of hyperspectral images are comparable and correspond well to qPCR results (see Fig. 4)

Figure 7 shows the results of severity level prediction for the VNIR camera. The severity level is estimated by the surface area which is classified as powdery mildew-affected. The results are presented from the aforementioned application perspective. The most relevant 3 cases are shown. First, segmentation results solely based on pixel-wise classification of the hyperspectral data are shown. In practice, this represents the default approach to hyperspectral image segmentation. The images have been grouped according to the expert’s decision about the infection level. For each of the groups of healthy, infected, and severely diseased grapes a boxplot of automatically estimated infection level is given in the upper diagram (A). While severely diseased biological material can be detected, detection of low infection states is not possible at a statistically significant level. Surprisingly, a Random Forest classifier cannot reliably handle the detection of healthy material as indicated by the mean offset for the estimated infection level if only normalized spectral data is used as feature vector. However, this is also related to the chosen training strategy. Training data comprised a sample from an independent set of two images from selected infected and healthy grapes. By recording the complete bunches, occlusions, shadows and blurring of image regions occur.

Given the same set of hyperspectral images, the proposed spatial-spectral segmentation of a projected hyperspectral image performs much better. Using LDA, a projection can be found which keeps the most relevant spectral information for the detection of powdery mildew infection. By calculating spatial features of the projected images a better discrimination between healthy bunches and bunches with only a few infected grapes is possible (middle diagram, B). Fig. 7c shows the improvements made by increasing the ensemble size to 50 random decision trees. Separation between healthy and infected bunches was further improved.

**Fig. 8:**
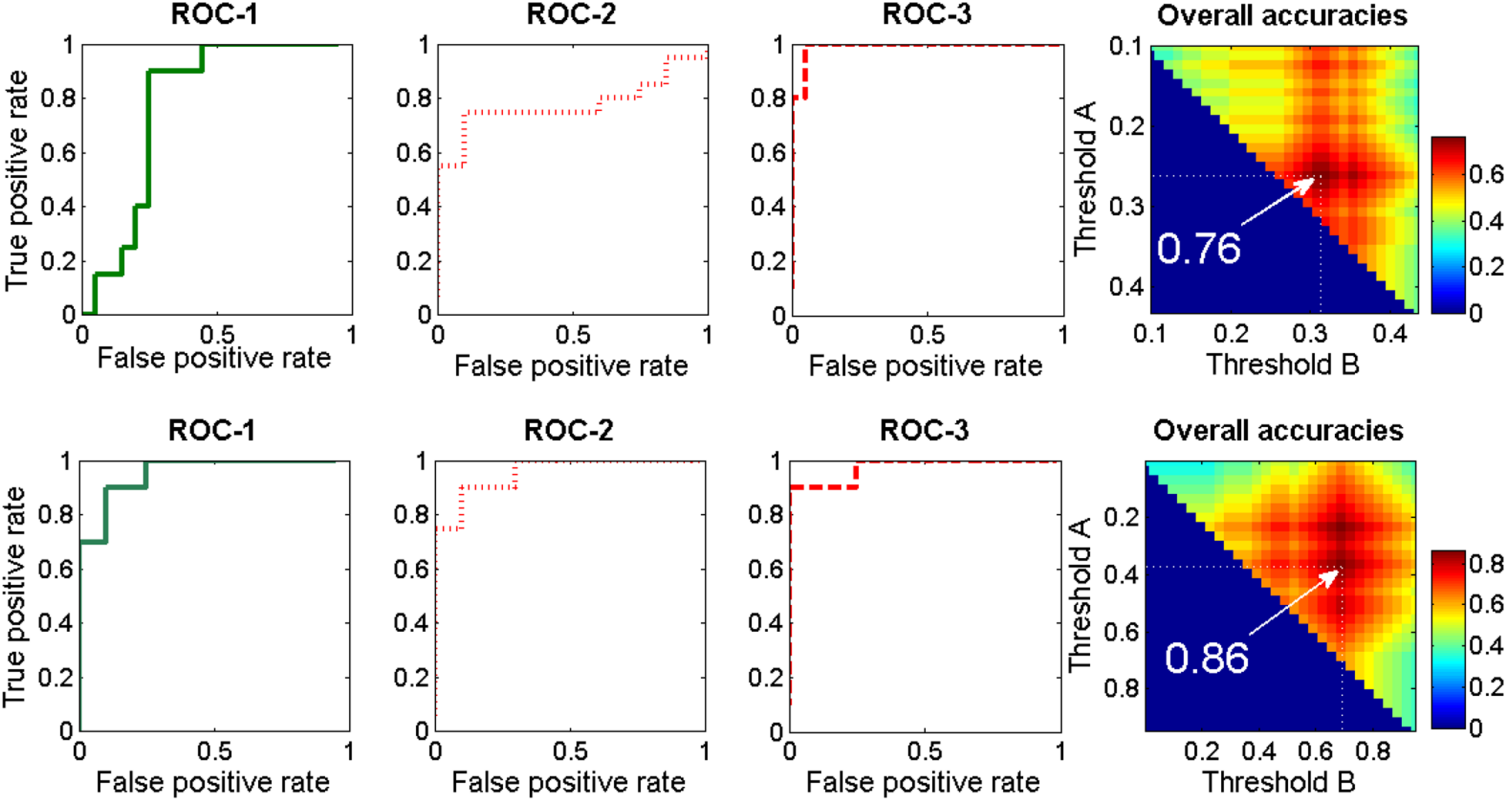
Receiver operating characteric curves and dependence of classification accuracy on selected thresholds. ROC curves visualize the trade-off between successful detection of healthy *versus* infected and severely diseased (ROC-1), infected and severely diseased *versus* healthy (ROC-2), and severely diseased *versus* all other bunches (ROC-3) and the corresponding error rates. ROC curves are calculated for the complete dataset of 60 images. Class decision for each bunch is based on the average fraction of diseased pixels of two images (*top* and *bottom view* of the bunch). This combined score was calculated for each bunch prior to application of a threshold. The *top row* shows the results for classification based on spectral features, while the *bottom row* shows the results for spatial-spectral features with Random Forest classifiers (50 trees each). A true positive rate of 1 means that all bunches of the corresponding class have been successfully assigned to the correct class. This is achieved at the price of a certain false positive rate, which denotes the fraction of bunches of the other classes falsely assigned to the same class. ROC-1 and ROC-2 are significantly improved by using spatial-spectral features. As two thresholds are needed to separate the 3 classes, the last column visualizes the accuracy as a function of the selected thresholds A and B. The optimal combination of thresholds is highlighted for both feature spaces and shows a significant gain in overall classification accuracy for our spatial-spectral approach

**Fig. 9:**
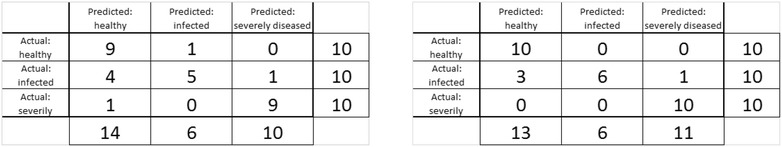
Classification results. Confusion matrices for thresholds corresponding to the operating points with maximum accuracy (see Fig. 8) of spectral (*left*) and spatial-spectral classification (*right*). For spatial-spectral classification, thresholds are found which allow perfect detection of healthy and severely diseased grape bunches. Also, the false detections of infected bunches as healthy and as severely infected are reduced by the spatial-spectral approach. The best automatically obtained decisions differ from visual assessment by experts only for 4 of the 10 infected bunches, with 3 classified as healthy and 1 classified as severely diseased. In addition, operating points can be adjusted according to application demands to provide a lower total accuracy but higher specificity/sensitivity for a certain class as needed

Figure 8 shows a different visualization of the classification performance for the complete dataset of 60 grape bunch images. Receiver Operating Characteristic (ROC) curves [40] are used to highlight the different trade-offs between true positive and false positive rates that exist for different threshold values. Thresholds are applied to the calculated fraction of diseased pixels to differentiate between healthy, infected, and severely diseased bunches. As the dataset contains two images of each bunch (top and bottom view), the mean of the two scores was calculated prior to application of thresholds. ROC curves and derived index values are often used for comparison of diagnostic tests [41] and can be used for optimal selection of operating points [42]. Diagrams ROC-1 correspond to the classification performances for the detection of healthy bunches *versus* overall infected (infected and severely diseased) based on spectral features (top row) and spatial-spectral features (bottom row), respectively. For each threshold the fraction of correctly classified healthy bunches is plotted against the false positive rate for the same threshold. For example, using spatial-spectral features a successfull detection of >80% of all healthy bunches (true positive rate >0.8) was achieved with a lower misclassification of infected bunches compared to using spectral features. This misclassification (error) directly corresponds to the contamination level when used for sorting a tranche of bunches. Diagrams in column ROC-2 show the inverse problem to separate any infected bunch (infected + severely diseased) from the group of healthy bunches. Obviously, in ROC-1 and ROC-2 diagrams the axes are exchanged. This illustrates the trade-off for the threshold-based decision, because the false positive rate now corresponds to the loss of healthy bunches (e.g. when the classifier is used in a sorting-machine). ROC-3 diagrams show the easier detection of severely diseased *versus* healthy and infected bunches. Both ROC-3 curves show that a higher fraction of severely diseased bunches can be detected with lower error compared to ROC-1 (healthy) and ROC-2 (overall infected). The last column shows the color coded classification accuracies as a 2-dimensional function of the thresholds for separating between the three classes (healthy, infected, severely diseased). The gain in classification accuracy for detection of infected bunches by using spatial-spectral features is clearly visible in diagrams ROC-1 and ROC-2, where the area under curve (AUC), which is related to classification accuracy, is increased. These improvements led to a significant gain in the overall classification accuracy from 0.76 (using only spectral data) to 0.86 (using spatial-spectral features). A detailed analysis of the performance gain is given in Fig. 9. The spatial-spectral approach significantly improves the ability to separate the three classes, especially for the difficult detection of infected bunches with little fungal biomass.

**Fig. 10:**
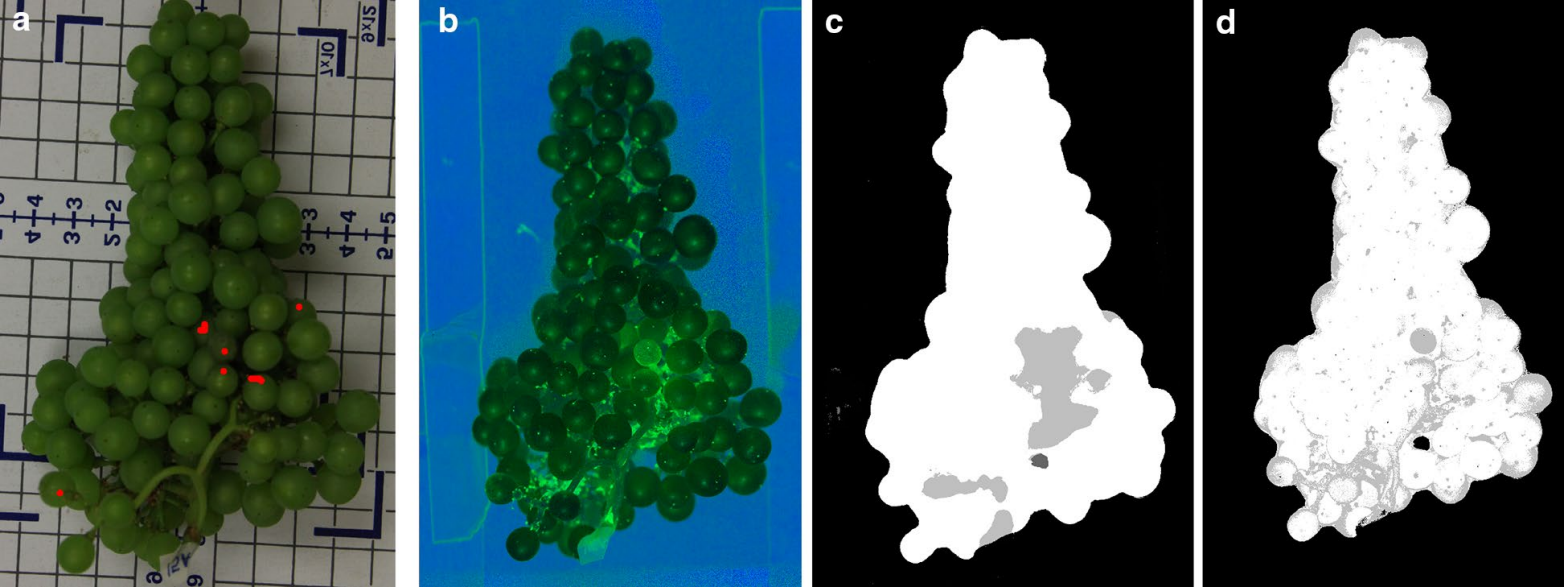
Visual representation of the results from the various data analysis approaches. Images of a representative scanned Chardonnay grape bunch: **a** example of a manually annotated grape bunch with visually identified infection sites shown as *red dots*, **b** disease specific visualization of VNIR hyperspectral image based on LDA coefficients, **c** powdery mildew detection results based on spatial-spectral approach (Table 1, row 8), **d** detection results based on classification of hyperspectral signatures (Table 1, row 1)

Figure 10 illustrates the general segmentation performance of the proposed method. The comparison with the manually annotated reference image highlights the capability of the Random Forest based segmentation approach to successfully detect powdery mildew affected grapes in VNIR hyperspectral images. Results for both spectral and spatial-spectral segmentation contain a number of pixels classified as false positive. As these pixels represent mainly background pixels which were not present in the original training dataset (detached berries only), the effect on the calculation of fractions of diseased/healthy pixels is comparable for all bunches of grapes. For this reason, we improved the approach by adding random samples from typical background regions (PTFE-plate, translation stage surface, paper labels, stem) of three additional hyperspectral grape bunch images to the training dataset. The pixels detected were then excluded from the count of diseased pixels. The accuracy values presented are based on the classification with background regions suppressed.

## Discussion

Hyperspectral imaging and data analysis based on spectral as well as spatial-spectral features have been applied here to test automated detection of powdery mildew infection of Chardonnay grape bunches within 12 h of routine in-field disease assessment. Hyperspectral imaging has already been used to develop spectral indices for detection of plant diseases [10], quantification of the spatial proportions within leaf lesions [43] and quantification of the intensity of sporulation and leaf colonization [9]. Several host-pathogen model systems, such as sugar beet and barley powdery mildew and grapevine leaf downy mildew, have been studied previously and, to our knowledge, we are first to report results for powdery mildew on grape bunches and individual berries in a controlled environment (Fig. 1). The approach presented in [10] requires exhaustive testing of the possible combinations of two wavelengths to find the best disease-specific index. Those indices (e.g. PSSR, PRI) along with a change of reflectance in particular spectral range are useful as they may indicate the degree of reaction of the disease-affected cells in the resistant and susceptible genotypes [44]. However, the use of only two wavelengths can be a major drawback and the incorporation of more wavelengths would drastically increase the amount of time required to find a solution. In our spatial-spectral approach, a disease-specific projection based on LDA is used instead. This approach can be easily transferred to any other model system. The main advantage is that the resulting projection is a linear combination of all wavelengths.

However, often the motivation for incorporating fewer wavelengths is to enable the application of simpler and cheaper sensor systems. For this it is important to identify the most relevant wavelengths from the hyperspectral dataset. In [10] the RELIEF-F algorithm is used prior to exhaustive testing to constrain the search space for the final solution for computational reasons. We have shown that similar information can be derived from the structure of the Random Forest classifier. We also showed for an adapted selection of three relevant wavelengths that a gain in classification accuracy (for detached berries) can be achieved when used in combination with textural features of image blocks instead of single pixels (Table 2). An alternative approach for identifying most relevant spectral features was reported in [45]. Here, Support Vector Machines (SVM) and Random Forest classifiers were coupled for classification of pine trees. An important aspect of this work was the utilization of Random Forest variable importance to identify the most relevant wavelength bands. Importance is based on ‘out-of-bag’ error and measures the average loss of accuracy when a single variable is not used. Experiments reported in [46] also include dimensionality reduction of hyperspectral data. The authors concluded that identifying the most relevant wavelength bands prior to classification yielded results similar to classification based on the complete spectral data. These findings showed that feature reduction was possible without significant loss of accuracy. An alternative approach to incorporate feature relevance into the training of Random Forest classifiers was proposed in [47]. Here, the randomness was induced in a guided way by selecting features based on a learned non-uniform distribution.

The promising results for intact bunches in the VNIR wavelength range and from cross-validation experiments within the training datasets (detached berries), in either the VNIR or SWIR domain, warrant further testing in a controlled environment and an industry setting to corroborate these findings. Results showed that a Random Forest with 50 random decision trees can be used to estimate infection and discriminate healthy bunches from infected. However, variation of hidden *E. necator* biomass and/or airborne conidia on the surface of berries in the visually healthy bunches indicates the need to set thresholds for characterization of healthy bunches.

The proposed algorithm for predicting powdery mildew severity needs to be validated in controlled conditions similar to those described by [48] for grape berries and bunches with intact conidia and during the latent period of *E. necator* development (i.e. between germination of the conidium and sporulation of the colony). This algorithm also needs to be validated using intact bunches harvested by hand at maturity, such as may be used for premium quality wines, small wineries, organic or biodynamic wines and dried products (e.g. raisins). Such validation will determine the sensitivity and precision of hyperspectral imaging under different conditions to assess its usefulness as a method to improve objective assessment of powdery mildew severity.

The proposed algorithm was developed for Chardonnay from a single vineyard at the beginning of bunch closure (E-L 30-33), when visually healthy and infected berries as well as the fungus differ in biochemical composition from that at harvest (E-L 38). Also, at harvest, skin and berry defects may be present due to biotic (e.g. other diseases and pests) and abiotic damage. It has been shown that LDA using data collected for berry color with an automated in-field phenotyping device (*PHENO*bot) could not predict red and rose berries if RGB values were used [49]. Consequently, it can be expected that additional adjustments, such as using grape bunches collected at harvest from a range of white and black grape varieties and growing regions, bunches with diverse compactness and those affected by other economically important diseases such as botrytis bunch rot [50], and validation in uniform light conditions, will improve the accuracy of hyperspectral imaging and prediction of powdery mildew severity on intact bunches. This approach may expand the application of hyperspectral discrimination of healthy and infected hand-harvested bunches in an industry setting. Implementation of hyperspectral imaging for sorting healthy and infected hand-harvested bunches in a single layer on a conveyor belt may be feasible.

Hyperspectral imaging has potential for real time assessment. However, substantial modification would be required to take into account differences between hand- and machine-harvested grapes. Machine-harvested grapes delivered to wineries comprise mainly individual detached and damaged berries plus material other than grapes (e.g. leaves, fragments of canes and vine bark). These detached berries can be either completely or partially covered with juice [51]. The presence of juice containing *E. necator* mycelia and conidia that are washed from the surface of infected berries during machine-harvesting might confound assessment due to reflection/scattering/shadow and the focal plane might differ from the recording of selected berries used for model generation. Therefore, classification models would need to be developed using detached berries covered with juice. High spatial resolution and variability within the juice-berry matrix make it necessary to define the most important characteristics of berry skin, where *E. necator* resides, to increase the reliability and sensitivity of the analysis. Consequently, sensitivity and accuracy of hyperspectral imaging will need to be tested in these conditions.

The qPCR results showed a need to establish thresholds for fungal biomass in visually healthy bunches and the same approach applies for hyperspectral imaging of those bunches. In the future, fungal biomass thresholds might be tentatively proposed for white and black varieties from different regions and validated through the perception of specific sensory characters in the resulting wine [32, 52].

## Conclusions

In this paper an approach to fast image segmentation has been adapted for segmentation of hyperspectral image data. Especially for automated plant phenotyping facilities, fast and robust algorithms are crucial for the analysis of imaging data from high-throughput experiments. Different dimensionality reduction methods have been tested to study the performance of spatial-spectral segmentation using Random Forest classifiers. The experimental results for the estimation of various powdery mildew infection levels on intact grape bunches show that the proposed spatial-spectral segmentation approach outperforms traditional pixel-wise classification of normalized spectral data by Random Forests. The use of a multiple classifier system, namely Random Forest, enables easy improvements in classification accuracy by increasing the ensemble size, fast feature extraction by calculating only the required features, as well as efficiency by parallel computation of the trees within the ensemble. Altogether, the application of the proposed image processing workflow has the potential to improve speed and accuracy in disease detection and monitoring in plant phenotyping applications. Also, it is applicable to all scales and, thus, will broaden the scope for the application of hyperspectral imaging technologies for the assessment of diseases, plant vitality, stress parameters, and nutrition status.
